# Rhythmic and sustained oscillations in metabolism and gene expression of *Cyanothece* sp. ATCC 51142 under constant light

**DOI:** 10.3389/fmicb.2013.00374

**Published:** 2013-12-06

**Authors:** Sandeep B. Gaudana, S. Krishnakumar, Swathi Alagesan, Madhuri G. Digmurti, Ganesh A. Viswanathan, Madhu Chetty, Pramod P. Wangikar

**Affiliations:** ^1^Department of Chemical Engineering, Indian Institute of Technology BombayPowai, Mumbai, India; ^2^Gippsland School of Information Technology, Monash UniversityVIC, Australia

**Keywords:** diazotrophic cyanobacteria, diurnal rhythm, *kaiC*, *nif*, RT-PCR

## Abstract

Cyanobacteria, a group of photosynthetic prokaryotes, oscillate between day and night time metabolisms with concomitant oscillations in gene expression in response to light/dark cycles (LD). The oscillations in gene expression have been shown to sustain in constant light (LL) with a free running period of 24 h in a model cyanobacterium *Synechococcus elongatus* PCC 7942. However, equivalent oscillations in metabolism are not reported under LL in this non-nitrogen fixing cyanobacterium. Here we focus on *Cyanothece* sp. ATCC 51142, a unicellular, nitrogen-fixing cyanobacterium known to temporally separate the processes of oxygenic photosynthesis and oxygen-sensitive nitrogen fixation. In a recent report, metabolism of *Cyanothece* 51142 has been shown to oscillate between photosynthetic and respiratory phases under LL with free running periods that are temperature dependent but significantly shorter than the circadian period. Further, the oscillations shift to circadian pattern at moderate cell densities that are concomitant with slower growth rates. Here we take this understanding forward and demonstrate that the ultradian rhythm under LL sustains at much higher cell densities when grown under turbulent regimes that simulate flashing light effect. Our results suggest that the ultradian rhythm in metabolism may be needed to support higher carbon and nitrogen requirements of rapidly growing cells under LL. With a comprehensive Real time PCR based gene expression analysis we account for key regulatory interactions and demonstrate the interplay between clock genes and the genes of key metabolic pathways. Further, we observe that several genes that peak at dusk in *Synechococcus* peak at dawn in *Cyanothece* and vice versa. The circadian rhythm of this organism appears to be more robust with peaking of genes in anticipation of the ensuing photosynthetic and respiratory metabolic phases.

## INTRODUCTION

Cyanobacteria are a group of photosynthetic prokaryotes that inhabit diverse ecosystems. They are believed to have played a role in conversion of the anoxic environment of earth to the present oxic one (Kasting, 2001). Cyanobacterial metabolism oscillates principally between day time metabolism including oxygenic photosynthesis and night time metabolism, of which some reactions may not be compatible with molecular oxygen (Liu et al., 1996; Kondo and Ishiura, 2000). It has now been established that these metabolic rhythms in cyanobacteria are controlled by a circadian clock rather than a real-time sensing of the light availability although the clock does get entrained by the external cues such as light and temperature. Thus, cyanobacteria are emerging as a model system for studies on circadian rhythms (Golden and Canales, 2003). Mechanism of cyanobacterial circadian clock has been experimentally enumerated in a model organism *Synechococcus* sp. PCC 7942 (henceforth *Synechococcus* 7942). The core clock comprises of three proteins KaiA, KaiB, and KaiC (Ishiura et al., 1998; Iwasaki et al., 1999). Of these, KaiC is the central oscillator that oscillates between its hyperphosphorylated and hypophosphorylated states at a frequency of 24 h, not just in vivo, but also in vitro when incubated with KaiA, KaiB, and ATP under appropriate conditions (Nakajima et al., 2005). The core clock proteins receive signals from the external environmental cues through the elements of the input pathway such as CikA, which is a histidine kinase and also known as a pseudobacteriophytochrome (Schmitz et al., 2000; Ivleva et al., 2006). As opposed to the initial belief, CikA has been found to sense the redox state rather than acting as a light absorbing photoreceptor (Ivleva et al., 2006). The output pathway is mediated through a sensory histidine kinase, SasA, and a transcription factor RpaA. While promoter activities and gene expression show a circadian rhythm, this does not translate into rhythmic oscillations in metabolism in *Syncechococcus* 7942. To exemplify, the photosynthesis rates oscillate in tandem with the promoter activity of *psbA1*, a key photosystem II gene, only in alternate light/dark (LD) but not in constant light (LL) conditions (Liu et al., 1995; Yen et al., 2004; Johnson et al., 2011).

In addition to the carbon cycle, cyanobacteria also play an important role in the nitrogen cycle via nitrogenase dependent fixation of atmospheric nitrogen (Sherman et al., 2010; Vijayan and O’Shea, 2013). The nitrogenase enzyme is irreversibly inhibited by oxygen, which necessitates mechanisms for its separation, either spatial or temporal, from the oxygenic photosynthesis in cyanobacteria (Dean et al., 1993). Unicellular nitrogen fixing cyanobacteria such as *Cyanothece* sp. ATCC 51142 (henceforth *Cyanothece* 51142) engage in night-time nitrogen fixation concomitantly with respiratory metabolism (Reddy et al., 1993; Schneegurt et al., 1997; Schneegurt et al., 2000; Alagesan et al., 2013; Bandyopadhyay et al., 2013). Respiration not only quenches oxygen but also supplies the energy required for nitrogen fixation by utilizing the glycogen granules stored during the day-time photosynthesis (Schneegurt et al., 1994). Microarray gene expression studies indicate that at least 30 and 10% of its genes exhibit circadian oscillations under LD (Stöckel et al., 2008) and LL (Toepel et al., 2008) conditions, respectively. In fact, some of the genes show an ultradian rhythm with two cycles of oscillations in a single diurnal period (Elvitigala et al., 2009). Further, culturing in a bioreactor has demonstrated sustained oscillations in optical density (OD) and levels of dissolved carbon dioxide and oxygen under LL (Červený and Nedbal, 2009). Recently, Červený et al. (2013) have shown ultradian rhythm in *Cyanothece* 51142 under continuous light. This and the other studies mentioned above, provide an excellent platform for the present study where we show how the oscillations in metabolism and gene expression in this organism are linked to the circadian clock components. We present a comprehensive real time PCR based study with representative genes from key metabolic pathways to demonstrate the interplay among them and their connection with the genes coding for the clock proteins. Červený et al. (2013) have demonstrated temperature dependence of ultradian rhythm under continuous light concluding that the ultradian rhythm may be independent of the circadian clock. They also propose that ultradian rhythm is conditional to low culture density and elevated CO_2_ requirements. In contrast, under our experimental set up of simulated flashing light effect (Krishnakumar et al., 2013), we observe rhythmic ultradian oscillations at gene expression and phenotypic level, which sustain even at higher cell densities and ambient CO_2_. Further, we observe that the transcription of the core clock genes and genes of the input and output pathway of the circadian clock, which oscillate at a frequency of 24 h under LD, reset their period to ~11 h under LL. This encourages us to believe that the ultradian rhythm may in fact be derived from the core circadian clock components and may indeed be the free running period for this organism, as exhibited both at gene expression as well as phenotypic level. We also bring out key differences in gene expression patterns of *Cyanothece* 51142 and *Synechococcus* 7942.

## MATERIALS AND METHODS

### CULTURE CULTIVATION AND SAMPLING

*Cyanothece* 51142 cultures were grown in an externally illuminated, air sparged and stirred photobioreactor in 1.7 L ASP2 medium without sodium nitrate (Reddy et al., 1993) at 30°C as described earlier (Krishnakumar et al., 2013) with a minor modification. We used an enhanced surface illumination of 230 μM photons in the present study. Exhaust gas profiles together with pH profile have been used to monitor the physiological state of the culture (Bapat et al., 2006; Maiti et al., 2009; Nigam et al., 2012; Krishnakumar et al., 2013). The online and offline parameters of the culture were monitored as described earlier (Krishnakumar et al., 2013). Briefly, the concentrations of carbon dioxide and oxygen in the exit gas from the photobioreactor were analyzed using BlueInOne Cell exit gas analyzer (BlueSens, Herten, Germany). pH of the growth medium was monitored with a pH probe (Hamilton, Bonaduz, Switzerland). Cell growth was monitored by measuring the OD of culture at 730 nm (OD_730_) using a Nanophotometer (Implen, München, Germany). Chlorophyll “a” was extracted by adding 1 ml methanol to cell pellets and heating at 60°C for 15 min and cooled to room temperature. Supernatant was collected after centrifugation at 12000 *g* for 15 min and absorbance was measured spectrophotometrically at 620, 678, and 750 nm (Arnon et al., 1974). Intracellular glycogen from the pellet obtained after chlorophyll extraction was hydrolyzed using 2N HCL (Reddy et al., 1993; Bandyopadhyay et al., 2010) and the released glucose was estimated using Glucose GOD PAP kit, a Glucose oxidase based enzyme assay kit (Biolab Diagnostics (I) Pvt. Ltd., Boisar, India). The culture was entrained under 12 h LD for 96 h followed by a free run under LL conditions. Nine samples were drawn from LD and 15 samples from LL for offline measurements such as growth, chlorophyll content, intracellular glycogen concentrations and for gene expression analysis. Samples were drawn from LD condition as described earlier (Krishnakumar et al., 2013). During LL, samples were drawn at times when the culture underwent transitions in its photosynthetic and respiratory phases, as indicated by the exit CO_2_ and O_2_ profiles. The data for concentrations of CO_2_ and O_2_ in exit gas was fitted to sinusoidal curves using cosine function Eq. 1 described earlier (Kondo et al., 1997) and using the optimize.curve_fit function of the SciPy library for Python.

(1) $$B=2^{-\left(t/D\right)}\,\left\{A\,\left[\cos\left(2\,\pi \,t/T+2\,\pi \,I/24\right)+1\right]+C\right\}$$

Where *B* is concentration of exit gas CO_2_/O_2_ in volume percent, *T* is period of circadian/ultradian rhythm, *D* denotes generation time (h), *A* denotes amplitude of the rhythmic component, *C* is a constitutive component, and I is the phase offset of the rhythm on a 24-h scale.

### RNA EXTRACTION AND GENE EXPRESSION ANALYSIS

For extraction of total RNA, samples were drawn at pre-determined intervals, centrifuged at 10000 *g* for 5 min at 4°C and 1 ml of pre-cooled TRI reagent (Sigma–Aldrich, St Louis, USA) was added and cells were stored at -80°C till further processing. The sample volume was adjusted with the varying culture density to use uniform quantity of biomass for RNA extraction. This was achieved by using sample volume such as the product of volume and OD_730_ was 10.0. Total RNA was extracted from the samples using TRI reagent (Sigma–Aldrich, St. Louis, MO) as described previously (Krishnakumar et al., 2013) with a minor modification which included the vortexing of thawed samples with 500 μm diameter acid washed glass beads (Sigma–Aldrich, St Louis, USA). We selected genes as representatives of photosystem I and II, nitrogenase complex, nitrogen regulation pathway, the clock comprising of *kai* genes, the input and output pathways of the clock, glycogen synthesis and degradation, carbon fixation, and cell division (**Table 1**). Gene expression analysis was performed using quantitative Real time PCR (qRT-PCR) analysis using LuminoCt SYBR Green qPCR ReadyMix (Sigma–Aldrich) on LightCycler^®^ 480 (Roche Diagnostics, Indianapolis, IN) and gene specific primers (**Table 1**) and as described earlier (Gaudana et al., 2013; Krishnakumar et al., 2013). The abundance in expression of individual genes was normalized with that of 16S rRNA gene as endogenous control and the equimolar pool of all RNA samples was used as control for calculating fold changes in expression as described earlier (Stöckel et al., 2008). The qRT-PCR data was clustered by hierarchical centroid linkage algorithm and euclidian distance measure using Cluster 3.0 open source software (de Hoon et al., 2004). The results of the clustering were visualized using a Java Tree view software (Saldanha, 2004).

**Table 1:** List of genes and the respective primer sequences used in the quantitative RT-PCR based gene expression analysis.

Gene symbol	Accession Id	Function	Primer sequences
*cikA*	cce_4751	Two-component hybrid sensor and regulator	F 5^′^cacctacgagtcacgggttt
			R 5^′^gacaatttgcccttgacgat
*kaiA*	cce_0424	Circadian clock protein	F 5^′^ggaaccctcgtacccgttat
			R 5^′^aacccgataacgcacttcag
*kaiB1*	cce_0423	Circadian clock protein	F 5^′^aattttaccccctcctgtcc
			R 5^′^tcgttttcccgttctttgat
*kaiB2*	cce_4715	Circadian clock protein	F 5^′^agcgatcgccaatctcttt
			R 5^′^ttttctcgttcggcctgtt
*kaiB3*	cce_0435	Putative circadian clock protein	F 5^′^ tccatcatctcctcgaaacc
			R 5^′^ ggtaggcgttgcagaaacat
*kaiB4*	cce_0145	Putative circadian clock protein	F 5^′^ gccatgaaatctacctattccc
			R 5^′^ tggcaatcatcgttaagatcc
*kaiC1*	cce_0422	Circadian clock protein	F 5^′^ gttcgcaaaattcggacaat
			R 5^′^ ttttcctgttcccgatgttc
*kaiC2*	cce_4716	Circadian clock protein	F 5^′^ acgaaatatgcgagggtttg
			R 5^′^ ccgttaccatcatcggttct
*rpaA*	cce_0298	Two-component response regulator	F 5^′^ cgggctttactcagacgaac
			R 5^′^ aaccaaatggcttcaaatcg
*sasA*	cce_1751	Adaptive-response sensory kinase	F 5^′^ aaaacaccccttttgcttca
			R 5^′^ aggtttccttcggcatcttt
*psaA*	cce_0989	Photosystem I P700 chlorophyll a apoprotein A1	F 5^′^ tgccaccctatccctaccag
			R 5^′^gggctccagcaccaactatt
*psbA1*	cce_3501	Photosystem II D1 protein	F 5^′^ atctttattctccgctatgc
			R 5^′^ tcttgtccgaacttgtaaccg
*rbcL*	cce_3166	Ribulose bisphosphate carboxylase	F 5^′^ tgccaccctatccctaccag
			R 5^′^gggctccagcaccaactatt
*glgA1*	cce_3396	Glycogen synthatase	F 5^′^ tcccgatcgcctaaaagata
			R 5^′^agcatggtgaggggatacag
*glpX*	cce_3974	Fructose 1,6-bisphosphatase II	F 5^′^ ctggaactttgatggaggga
			R 5^′^ gtatcaacgaaacgggctgt
*glgP1*	cce_1629	Glycogen phosphorylase	F 5^′^ ccattggttatggtatccgc
			R 5^′^ caggggttctcaaaccgtaa
*ccmK2*	cce_4283	Carbon dioxide concentrating mechanism protein	F 5^′^ cgtgttacggttattgtgcg
			R 5^′^ gtcgatagaacctctcccc
*nifH*	cce_0559	Nitrogenase iron protein	F 5^′^taccattgctgcgttagctg
			R 5^′^gtgcagaatggtggtttgtg
*nifX*	cce_0565	Nitrogen fixation protein	F 5^′^gacccccattaaagcgagaa
			R 5^′^ttaaccaaggaggcggattt
*ntcA*	cce_0461	Nitrogen-responsive regulatory protein	F 5^′^aattttaccccctcctgtcc
			R 5^′^tcgttttcccgttctttgat
*PatB*	cce_1898	Cytochrome b6, transcriptional regulator for nitrogen fixation	F 5^′^agcgatcgccaatctcttt
			R 5^′^ttttctcgttcggcctgtt
*cphA*	cce_2237	Cyanophycin synthetase	F 5^′^ tgatcacctggggttaggag
			R 5^′^ taagcactgcgtaaccatcg
*ftsZ*	cce_1314	Cell division protein	F 5^′^ gatgaacgggttcaaggaga
			R 5^′^ ttaggggttgaggtgctttg
*rrn16Sa*	cce_RNA045	Constitutive expression	F 5^′^ ccctgggctacacacgtact
			R 5^′^ tctcgagttgcagagagcaa

*This includes genes coding for core clock proteins, proteins involved in input and output pathways of circadian clock and select genes coding for proteins involved in key metabolic pathways.
*

## RESULTS AND DISCUSSION

### OSCILLATIONS IN METABOLISM

**FIGURE 1:**
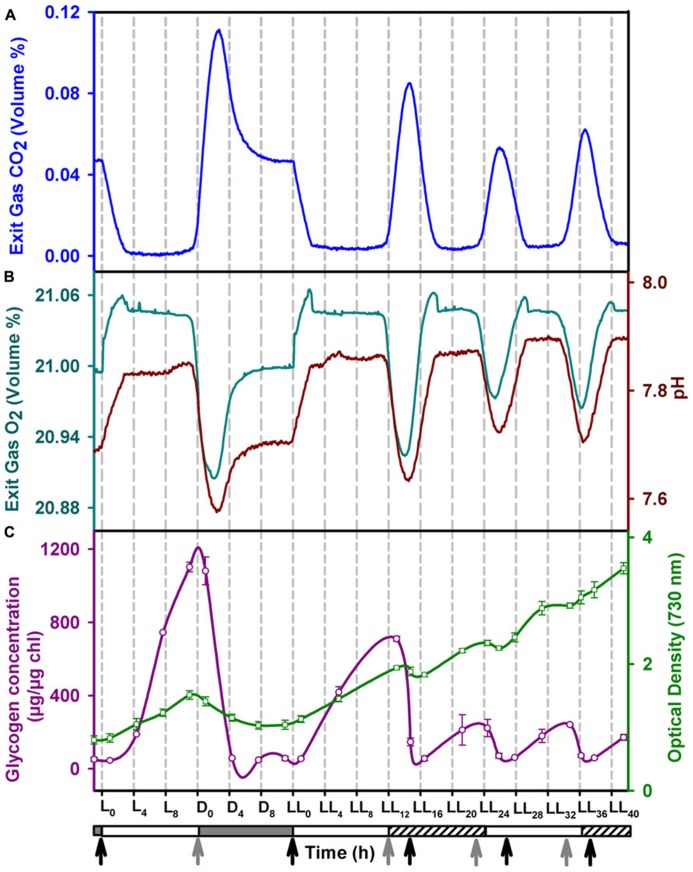
**Profiles of (A) CO_2_ and (B) O_2_ in the exit gas (cyan), pH of the growth medium (brown), **(C)** intracellular glycogen (red) content and growth (green) in the fourth day of entrainment under alternating light/dark cycles (LD) followed by constant light (LL)**. The horizontal bar below the *X*-axis denotes the light (clear), dark (shaded) and subjective dark (shaded with slanted lines) phases. The onset of photosynthetic and respiratory phases are marked with black and grey arrows, respectively. Error bars on the glycogen and OD data points denote ± SEM for *N* = 3.

**FIGURE 2:**
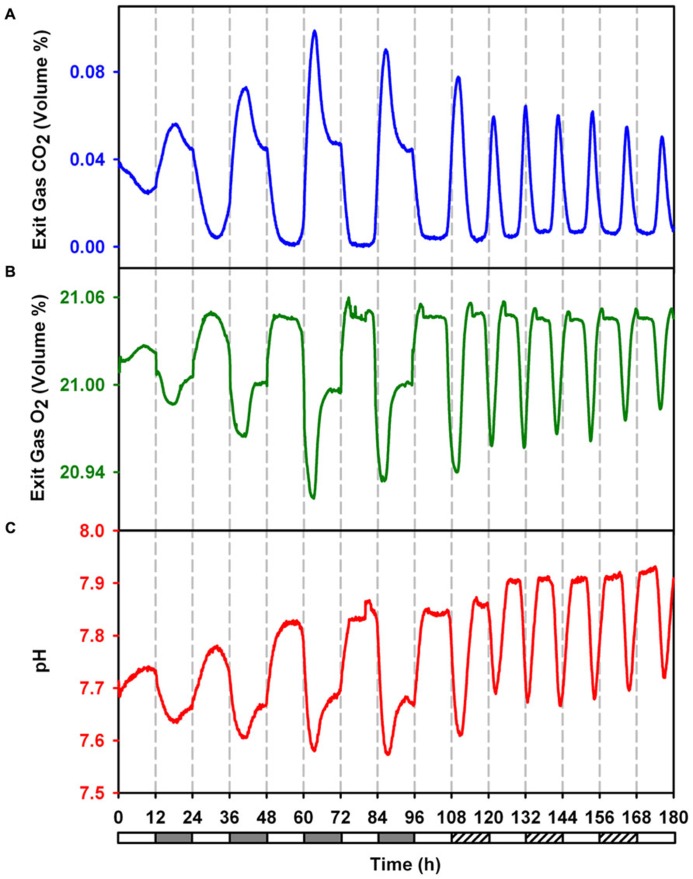
**Online monitored exit gas and growth medium pH profiles during 4 days LD followed by 3 days in LL to demonstrate sustained ocillations in metabolism during free run under constant light. (A)** Concentration of CO_2_ in exit gas. **(B)** Concentration of O_2_ in exit gas. **(C)** pH of growth medium.

Under LD conditions, respiratory bursts are observed around the light switch-off time and once every 24 h (**Figures 1A,B** and **2**). We observe a respiratory burst at approximately 12 h into constant light (LL_12_), which lasts for about 2 h followed by a photosynthetic phase. This is also approximately 24 h after the last respiratory burst in the LD phase. Subsequent respiratory bursts were observed at intervals of ~ 11 h with intervening photosynthetic phases. Respiratory bursts are typified by net CO_2_ evolution, net O_2_ consumption and a drop in pH and last for about 2 h. The oscillations sustained for at least 84 h into LL, the duration for which data was collected (**Figure 2**). In the present study, we used surface illumination of 230 μM photons, an optimal value for the current vessel geometry and hydrodynamic conditions. This is in contrast to earlier reports where surface illumination of 50–90 μM photons has been used (Colón-López and Sherman, 1998; Červený and Nedbal, 2009; Krishnakumar et al., 2013). The turbulent regime ensures flashing light effect thereby alleviating potential artifacts arising out of light limitation or light inhibition.

The data for CO_2_ and O_2_ concentration in the exhaust gas was fit in sinusoidal curves which denoted a period of 23.26, 10.65, 23.47, and 10.54 h for CO_2_ under LD, CO_2_ under LL, O_2_ under LD and O_2_ under LL respectively. The ultradian rhythm between the alternate cycles of carbon and nitrogen fixation in this organism principally seems out of the requirement to meet the demand of high levels of both carbon and nitrogen for rapidly growing cells under LL condition.

Nighttime biomass loss (NBL) is a well known phenomenon in nitrogen fixing unicellular cyanobacteria (Ogbonna and Tanaka, 1996; Carlozzi, 2003). This is usually ascribed to decrease in intracelluar glycogen content (Ogbonna and Tanaka, 1996). We observe NBL not only during LD conditions but also during LL conditions coinciding with the respiratory bursts. The intracellular glycogen content oscillates reaching a peak value just before the respiratory bursts during LL conditions. While NBL coincides with the decrease in glycogen content, the amplitudes of glycogen and biomass oscillations are smaller in LL condition than those in LD conditions (**Figure 1C**).

### OSCILLATIONS IN GENE EXPRESSION

**FIGURE 3:**
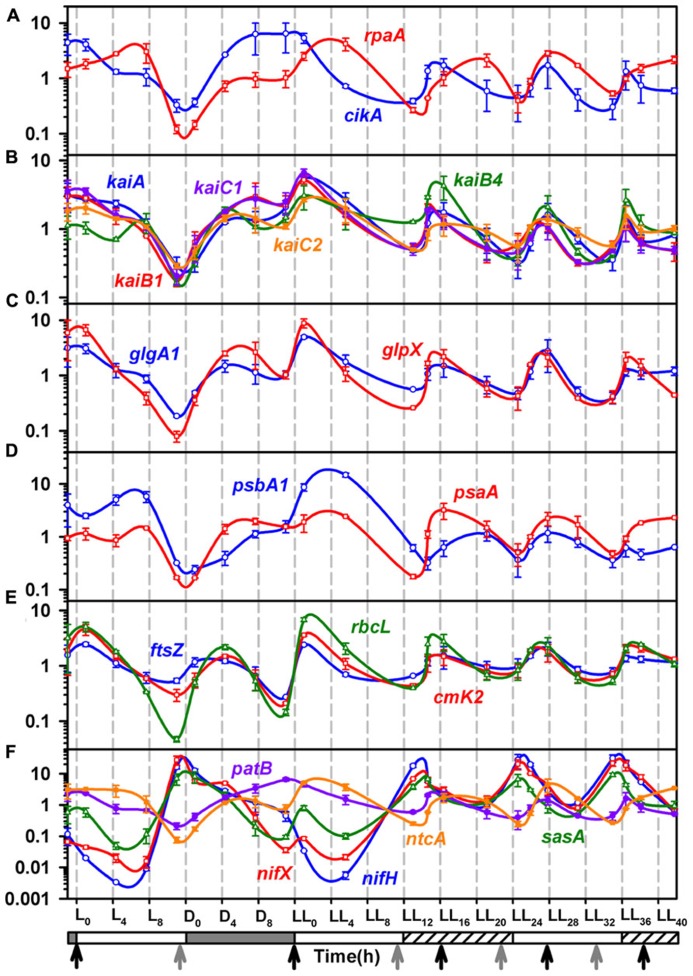
**Quantitative Real-time PCR analysis for total RNA samples from the 4th day of entrainment under alternating light/dark cycles (LD) followed by constant light (LL).(A)** The level of transcripts for *cikA* (blue), and *rpa* (red), involved in input and output pathway and output pathway of circadian rhythm; **(B)** The trasncription profiles for clock genes, *kaiA* (blue), *kaiB1* (red), kaiB4 (green), *kaiC1* (violet) and *kaiC2* (orange); **(C)** The expression profiles of glycogen synthesis genes, *glpX* (red) and *glgA1* (blue) matches closely with that of *kai* genes, indicating a possible close association of glycogen synthesis with the clock. **(D)** The transcript profiles of *psbA1* (blue) and *psaA* (red), genes of Photosystem I and II respectively; **(E)** The epxpression profiles of genes involved in carbon fixation, *rbcL* (green) and *ccmK2* (red) and cell division, *ftsZ* (blue); **(F)** Transcription profiles of the circadian rhythm output pathway gene *sasA* (green) and genes of nitrogenase complex, *nifH* (blue) and *nifX* (red), nitrogen fixation regulation genes *patB* (violet) and *ntcA* (orange). Refer to **Figure 1** for other details and **Table 1** for details of genes and PCR primers.

**FIGURE 4:**
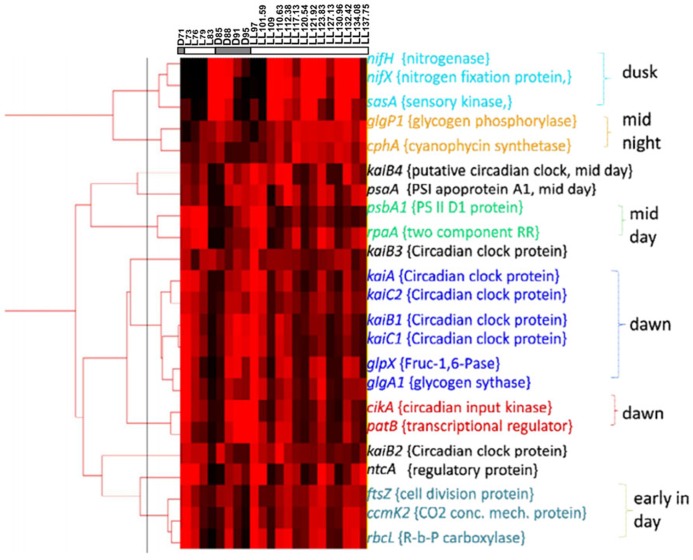
**Clustering analysis on basis of the Real time PCR expression data of genes coding for clock genes, input and output pathway of circadian rhythm and selected representatives from genes involved in various metabolic pathways**.

Stöckel et al. (2008) have reported rhythmic changes in expression of ~ 30% of the genes in response to LD cycles. A large number of metabolic genes showed oscillations suggesting prevalence of differences between metabolic activities that occur under the light and the dark periods. The rhythm sustained in some of the genes for the first 24 h into LL (Toepel et al., 2008). Therefore, it was of interest to see if gene expression shows rhythmic changes under LL beyond the first 24 h and if these oscillations correlate with those in metabolism (**Figure 3**). The results, in general, are in agreement with the microarray gene expression results obtained for two diurnal cycles (Stöckel et al., 2008) and for a diurnal cycle followed by constant light for 24 h (Toepel et al., 2008). Some genes such as *psaA*, *rbcL*, *cikA*, *rpaA*, *nifX,* and *nifH*, show a delayed response in the microarray results (Stöckel et al., 2008; Toepel et al., 2008). These may be either due to the differences in cultivation conditions, the techniques of gene expression studies or sampling frequency or a combination thereof. Interestingly, gene expression pattern of *patB* is out of phase with that of both the microarray studies. Clustering of our gene expression data (**Figure 4**) shows that the genes can be broadly categorized as dawn-peaking and dusk-peaking (**Figures 3A–F** and **4**) which is consistent with earlier report (Stöckel et al., 2008). Majority of the genes tested under the present study oscillate at a frequency of 24 h under LD and a frequency of about 11 h under LL condition with a few exceptions. The genes *ftsZ*, *ccmk2,* and *rbcL* peak twice in a 24-h period, even under LD (**Figure 3E**). This has been reported earlier from microarray gene expression data for a few other genes (but not for *ftsZ*, *ccmK2,* and *rbcL* genes) and such genes were designated as those with ultradian rhythm (Elvitigala et al., 2009).

### OSCILLATIONS IN GENES OF PHOTOSYSTEM I AND II

The genes *psaA* and *psbA1* are generally used as representatives of the photosystem I and II, respectively (Colón-López and Sherman, 1998). In fact, the *psbA* promoter has been shown to oscillate in *Synechococcus* 7942 in constant light with a free run period of 24 h (Kondo et al., 1993). Further the *psbA1* expression and protein levels have been shown to be up-regulated for about 2/3rd of the light phase in LD in *Cyanothece* 51142 (Colón-López and Sherman, 1998). While the *psaA* and *psbA1* genes peak (and bottom out) once every 24 h under LD conditions (**Figure 3D** and Colón-López and Sherman, 1998), they oscillate at intervals of ~11 h under LL conditions. The peaks coincide with late respiratory phase thereby preparing the photosynthetic machinery for the ensuing photosynthesis phase. Likewise, *ccmK2*, the gene involved in carbon concentrating mechanism, *rbcL*, the gene for large subunit of Rubisco enzyme and *fts*Z, a gene involved in cell division, oscillate in tandem with *psaA* and *psbA1* under LL condition (**Figure 3E**). Thus, the entire machinery gets upregulated and ready before the photosynthetic phase.

### OSCILLATIONS IN GENES OF NITROGEN METABOLISM

The respiratory bursts in LD and LL are preceded by peaks in *nifH* and *nifX* genes indicating that the cells gear up for nitrogen fixation which needs to be synchronized with the respiratory burst. Under LD, *nif* genes peak at time point L_11_, this is an hour before the onset of dark (**Figure 3F**). Prior studies have shown peaking of *nif* genes only after the onset of dark with steady levels of transcript through the night under LD conditions (Stöckel et al., 2008). On the other hand, we observe complete degradation of the *nif* gene transcripts through the night after the pre-dusk peak. This suggests that the culturing conditions may have a bearing on the diurnal rhythm of the organism. Under LL, we observe rhythmic oscillations in the *nif* genes with a period of ~11 h, similar to that for the photosynthesis genes as well as the physiological parameters measured in terms of exit gas and media pH profiles as well as intracellular glycogen content. The expression profiles of *ntcA* and *patB*, the global regulators for nitrogen fixation (Kolodny et al., 2006; McDermott et al., 2011), are out of phase with that of *nif* genes (**Figure 3F**). Indeed, the expression of *ntcA* has been reported to be out of phase with that of the *nifHDK* operon under nitrogen fixing conditions for a *Cyanothece* strain (Bradley and Reddy, 1997).

### OSCILLATIONS IN GENES OF CIRCADIAN RHYTHM

Molecular mechanism for the circadian clock and its input and output pathway has been extensively reported for *Synechococcus* 7942. The redox state of intracellular quinone pool is a key factor in the signaling of switch from daytime to nighttime activities (Kim et al., 2012). The intracellular plastoquinone (PQ) pool stays in a reduced state during the light phase with a sudden drop in the reduced pool at the onset of dark (Kim et al., 2012). Only the oxidized form of quinone binds to CikA as well as KaiA and induces the degradation of CikA and aggregation of KaiA (Ivleva et al., 2006; Kim et al., 2012). CikA then induces *rpaA* and RpaA then dislocates RpaB from the *kaiBC* operon leading to its induction (Hanaoka et al., 2012).

**FIGURE 5:**
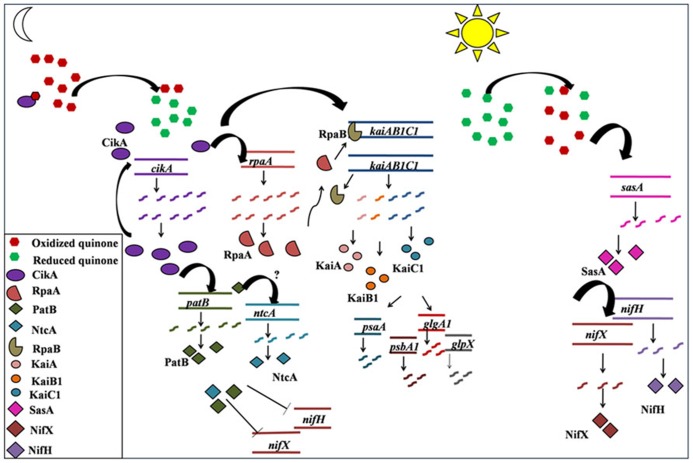
**A schematic representation of the proposed sequence of transcription events for temporal separation in *Cyanothece* sp. ATCC 51142**. The predictions are based upon the transcription profiles of genes monitored using RT-PCR in the present study and on the existing understanding of the functioning of different components of circadian rhythm in *Synechococcus* sp. PCC 7942. Reduction of PQ pool, following the respiratory burst at dusk, leads to activation of CikA (Kim et al., 2012), which inturn activates its own transcription as well as the transcription of *kaiAB1C1*cluster, possibly via RpaA as reported for *Synechococcus* 7942 (Hanaoka et al., 2012) or by an alternate pathway. The transcription and translation of *kaiAB1C1* cluster in turn leads to initiation of transcription of genes for photosystem I and II as well as for glycogen synthesis, all of which peak at dawn in anticipation of the ensuing light phase. Oxidation of PQ pool possibly activates transcription of *sasA.* SasA activates the transcrition of *nif* genes which peak at dusk and prepare the system for carrying out nitrogen fixation during the respiratory burst.

While homologs of many of the clock genes are present, the regulatory interactions have not been enumerated in other cyanobacteria including *Cyanothece* 51142. There are some notable differences in the gene expression patterns of *Synechococcus* 7942 (Ito et al., 2009) and *Cyanothece* 51142. The genes of the circadian clock and the input and output pathway oscillate at a frequency of ~11 h in *Cyanothece* 51142 as compared to that of 24 h in *Synechococcus* 7942 under constant light condition. Further, the genes for KaiA, KaiB, KaiC, and CikA homologs in *Cyanothece* 51142 peak at dawn (**Figures 3A,B**) in contrast to their peaking at dusk in *Synechococcus* 7942 (Kondo and Ishiura, 2000; Vijayan and O’Shea, 2013). While the gene regulatory interactions may have been conserved between *Synechococcus* 7942 and *Cyanothece* 51142, we propose that the phase differences may originate from the redox state of the PQ pool mediated by glycogen metabolism. We propose that in *Cyanothece* 51142, the reduced quinone pool declines gradually during the day as a result of glycogen synthesis with a burst in the reduced pool triggered by the respiratory burst at dusk (**Figure 5**). This might be one of the mechanisms responsible for oxidation of the intracellular quinone pool during the light phase. This is in agreement with the prevailing understanding about the intracellular storage molecules such as glycogen being instrumental in influencing the redox state of the intracellular quinone pool (Kreysa and Krämer, 1989; Sherman et al., 2010). Previously it is shown that glycogen is a key respiratory substrate and that an impaired glycogen synthesis negatively influences metabolic regulations in another cyanobacterium *Synechocystis* sp. PCC 6803 (Gründel et al., 2012). A direct interaction of glycogen synthase with thioredoxin in *Synechocystis* 6803 was also recently proposed (Díaz-Troya et al., 2013). Further, we believe that the role of glycogen could be analogous to that played by starch in *Arabidopsis*, in which the intracellular concentrations of solutes such as glucose and sucrose play a central role in providing a feedback to the circadian clock genes, though the mechanism for this feedback is not understood (Haydon et al., 2011). Thus, glycogen synthesis and degradation processes in *Cyanothece* 51142 are likely to play a key role in regulating the circadian rhythm on two counts: by dictating the concentration of intracellular glucose and by acting as respiratory substrate. However, further direct experimental evidences are required to validate this hypothesis.

In *Cyanothece* 51142, there is no noticeable phase lag between the expression of *rpaA* and the *kaiAB1C1* cluster (**Figures 3A,B**), suggesting that the signal from CikA may be conferred to the *kaiAB1C1* cluster by an alternate intermediate other than RpaA. The initiation of transcription of the *kaiAB1C1* cluster then leads to the transcription of genes involved in daytime activity such as photosynthesis (*psbaA*, *psbA1*) and glycogen synthesis (*glpX*, *glgA1*; **Figures 3C,D** and **5**). The gradual oxidization of the quinone pool during the photosynthesis phase, as result of the glycogen synthesis, might be the signal conferred to the *nif* genes through SasA (**Figures 3F** and **5**).

During the first 2 h into the first subjective dark phase under LL, the chronology of events remains similar to that as the first 2 h of the dark phase under LD. The intracellular glycogen concentration falls and so does the exit gas O_2_ and the pH. However, probably because of the presence of light even during the subjective dark might have caused an early reduction of the quinone pool, since the redox state of the quinone pool is understood to be controlled by the photosynthetic electron transport as a function of light availability and by the respiratory electrons in dark (Kim et al., 2012). This could in turn have shifted the rhythm into the day time activity such as photosynthesis and glycogen synthesis. The same is indicated by the increase in the intracellular glycogen concentration in the middle of the first subjective dark phase unlike that under the dark phase of LD, where glycogen concentration remains at the base till the initiation of the light phase. This is also synchronous with the upregulation of the phostosynthesis genes *psbA1* and *psaA*, glycogen synthesis genes *glgP1* and *glgX* and the *kai*A, *kaiB1*, *kaiC1,* and *kaiC2* gene cluster, which all peak at the 4–6 h of the first subjective dark and then at a frequency of 11 h then onward. The beginning of the first subjective dark leads to the peaking of the *nif* genes and that of intracellular glycogen concentration. These peaks are then observed at a frequency of about 11 h and in tandem with the above described day time activities such as photosynthesis and glycogen synthesis.

### COMPARISON OF CIRCADIAN RHYTHM OF *Cyanothece* 51142 AND *Synechococcus* 7942

*Synechococcus* 7942 exhibits a free run period of 24 h with its doubling time of 6 h (Mori et al., 1996) while *Cyanothece* shows a free run period of ~11 h and a doubling time of 30 h in constant light under the present culturing conditions. This shows that there may not be a correlation between the doubling time and free run period in cyanobacteria. Further, there are some noticeable differences between the two organisms in the terms of the genes of the clock and the output pathway. Specifically, the *kaiAB1C1* operon found in *Cyanothece* 51142 (Memon et al., 2013) is shorter by one gene and present as *kaiBC* in *Synechococcus* 7942 (Kutsuna et al., 2005). While *Synechococcus* 7942 contains only one gene each for KaiA, KaiB, and KaiC, *Cyanothece* 51142 contains multiple homologs of KaiB and KaiC (**Table 1**). Importantly, homolog of the period extender protein Pex (Kutsuna et al., 1998; Tanaka et al., 2012) is missing in *Cyanothece* 51142. However, lack of the *pex* gene may only partially explain differences in the free run periods of the two organisms. Further, the photosystem genes such as *psbA1* and *psaA* peak at dawn and stay upregulated for about 2/3rd of the day in *Cyanothece* 51142 (**Figure 3D**) in contrast to the peaking at dusk in *Synechococcus* 7942 (Kondo and Ishiura, 2000; Vijayan and O’Shea, 2013). Likewise, the *nifH* and *nifX* genes peak pre-dusk in anticipation of the ensuing respiratory burst (**Figure 2F**), which is necessary for the nitrogen fixation activity. On the whole, the main contrast between the circadian rhythm of *Synechococcus* 7942 and *Cyanothece* 51142 seems to be based upon the robustness that enables *Cyanothece* 51142 to be prepared for the next metabolic phase during the existing metabolic cycle while it temporally regulates the separation of oxygenic photosynthesis from oxygen sensitive nitrogen fixation process.

The present study, which was conducted under prolonged constant light, provides understanding about the physiological and molecular behavior of *Cyanothece* 51142 under constant light and is a concrete evidence of a circadian rhythm in and temporal separation of key metabolic processes under this free running condition.

## Conflict of Interest Statement

The authors declare that the research was conducted in the absence of any commercial or financial relationships that could be construed as a potential conflict of interest.

## Author Contributions

Sandeep B. Gaudana and S. Krishnakumar have contributed equally to this work. Sandeep B. Gaudana, S. Krishnakumar and Pramod P. Wangikar designed research; Sandeep B. Gaudana, S. Krishnakumar, Swathi Alagesan and Madhuri G. Digmurti performed research; Sandeep B. Gaudana, S. Krishnakumar, Madhuri G. Digmurti, Ganesh A. Viswanathan, Madhu Chetty and Pramod P. Wangikar analyzed the data; Sandeep B. Gaudana and Pramod P. Wangikar wrote the paper.
